# Long‐term disease control and survival observed after stereotactic ablative body radiotherapy for oligometastatic breast cancer

**DOI:** 10.1002/cam4.4068

**Published:** 2021-06-22

**Authors:** N. Ari Wijetunga, Carlos H. dos Anjos, W. Iris Zhi, Mark Robson, C. Jillian Tsai, Yoshiya Yamada, Laura Dover, Erin F. Gillespie, Amy J. Xu, Jonathan T. Yang

**Affiliations:** ^1^ Department of Radiation Oncology Memorial Sloan Kettering Cancer Center New York NY USA; ^2^ Department of Medicine Division of Solid Tumor Oncology Breast Medicine Service Memorial Sloan Kettering Cancer Center New York NY USA; ^3^ Precision Radiation for Oligometastatic and Metastatic Disease (PROMISE) Program Department of Radiation Oncology Memorial Sloan Kettering Cancer Center New York NY USA

**Keywords:** breast cancer, metastasis, oligometastases, oncogenomics, radiation therapy, women's cancer

## Abstract

**Purpose:**

We examined the characteristics of breast cancer patients with oligometastases (OM) treated with stereotactic ablative body radiotherapy (SABR) to identify factors associated with local progression, distant metastasis progression, time to subsequent therapy, progression‐free survival (PFS), and overall survival (OS).

**Methods:**

We retrospectively reviewed a single‐institution database of patients treated with radiotherapy between 2008 and 2018 and identified 79 patients who received SABR to OM. Twenty‐seven patients had genetic testing of metastatic tumors using an institutional targeted sequencing platform. Kaplan–Meier analysis, Cox regression, and competing risk models were used to compare clinical and genetic correlates with outcomes.

**Results:**

Median follow‐up was 50 months (IQR: 29–66) with 67% of patients alive at the last follow‐up. Of the 65% of patients who progressed, 82% progressed outside of the radiation field, 18% experienced local failure, and 80% had oligoprogression. Median OS was 86 months (IQR: 29–66), and PFS was 33 months (IQR: 10–38). Less than 5 years from diagnosis to SABR and triple‐negative breast cancer (TNBC) were associated with worse OS. Advanced T stage, any prior chemotherapy, and TNBC were associated with worse PFS. Alterations in CEBPB, RB1, TBX3, PTEN, and CDK4 were associated with worse survival outcomes.

**Conclusion:**

Long‐term systemic disease control and survival can be achieved with SABR for oligometastatic breast cancer. Hormone receptor‐positive patients with a long disease interval from initial diagnosis and limited systemic progression history may be ideal for SABR to all sites of disease.

## Graphical abstract

Through a retrospective review of our institution's oligometastatic breast cancer patients who were treated with stereotactic ablative radiotherapy, we identified clinical and genetic correlates with survival outcomes, including overall survival and progression‐free survival. Our work substantiates the role of high dose targeted radiotherapy in metastatic breast cancer patients with a low burden of disease, and our findings provide insight into optimal patient selection and elucidate clinical outcomes.

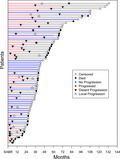

## 1 INTRODUCTION

Metastatic breast cancer presents at the time of diagnosis in approximately 6% of breast cancer patients, and it develops in 20%–50% of breast cancer patients during their lifetimes.^1^ Survival for these patients is improving, reflecting improved therapies and an aging population.^2^ The greatest benefits in survival are seen in the hormone receptor‐positive breast cancers, likely due to the widespread use of hormonal therapy in these patients.^3^ As a result, survivorship in metastatic breast cancer patients can vary greatly by breast cancer subtype. By 2020, there are projected to be at least 170,000 women in the United States living with metastatic breast cancer,^2^ and the optimal therapy to control metastatic disease remains unclear.

Historically, women with metastatic breast cancer were treated with systemic therapies, while local therapy was reserved for palliation only. It is now recognized that there exists a subset of patients for whom local therapy, such as radiotherapy (RT), may be a vital component to treatment. In particular, patients with metastatic breast cancer with a relatively low burden of metastatic disease, or oligometastases (OM), account for up to 20% of all metastatic breast cancer patients and may have a better prognosis after local therapy compared to patients with a high metastatic disease burden.^4^

There is a growing body of research suggesting that stereotactic ablative body radiotherapy (SABR) is an effective local treatment for OM. In 2007, the European School of Oncology Metastatic Breast Cancer Task Force called for clinical trials evaluating the efficacy of more aggressive local therapy and a multidisciplinary approach in the setting of oligometastatic breast cancer.^5^ Subsequently, delivering high dose per fraction radiation through SABR was demonstrated in several randomized trials to have a survival benefit when incorporated into the management of patients with oligometastatic lung cancer, though radiotherapy‐related toxicities have been observed.^6^, ^7^, ^8^ For patients with breast cancer, who comprised a subset of patients evaluated on SABR‐COMET, significant improvement in overall survival was observed when oligometastases were treated SABR.^8^

There is an increasing frequency of SABR use in the management of patients with OM but limited long‐term retrospective and prospective evidence regarding outcomes or safety at this time.^9^ The NRG Oncology group trial BR001 phase I trial showed that it was safe to treat up to four metastatic sites with SABR in seven different anatomical locations.^10^ Currently, the BR002 phase II randomized study is evaluating the standard of care including systemic therapy and palliative radiotherapy compared to standard of care with SABR for OM breast cancer, but the study is on hold for reporting its results given the low frequency of events.^11^

The prospect of treating OM has the potential to shift treatment goals from the quality of life improvement to survival benefits and disease remission.^12^ In this context, we set out to examine our institutional experience on the clinical outcomes and associated clinical and molecular factors using strict inclusion criteria, long follow‐up time, and characterization of disease progression in breast cancer patients who underwent SABR for oligometastatic breast cancer.

## 2 METHODS

### 2.1 Study population

Institutional Review Board approval was obtained for this study. We conducted a retrospective review of patients with metastatic breast cancer who received radiotherapy between 2008 and 2018 and identified 1,936 patients, of whom 393 patients received SABR for metastatic disease. We examined a further subset of 79 patients with 103 lesions who (a) had biopsy‐confirmed metastatic disease, (b) had OM with ≤5 extracranial metastases, (c) had no prior RT for extracranial metastatic disease, and (d) received SABR (BED_4_ greater than or equal to 60 Gy) to all known extracranial metastases at the time of treatment. Only one patient had prior stereotactic radiosurgery for intracranial metastases. Radiation treatment to metastatic sites within 30 days of one another was considered part of the same treatment course. All censored patients had at least 1 year of follow‐up. Clinical information was obtained including patient demographics, pathologic staging of the primary disease (Table S1), hormone receptor (HR) positivity, HER2 positivity, site of oligometastasis (Table S2), pre and post‐RT systemic therapy use (Tables S4 and S5), time from breast cancer diagnosis to SABR, number of sites treated at RT (Table S3), RT dose (Table S6), symptoms pre and post‐RT, and toxicity of RT. We determined at the time of SABR whether the type of oligometastatic disease was newly diagnosed metastatic disease, stable metastatic disease on systemic therapy for at least 3 months, or progression of a known oligometastatic lesion. Clinical outcomes (Table S7) for patients who progressed included the site of progression, whether patients had oligoprogression (progression at fewer than five sites), and how progression was treated. Patients who did not die were censored at the study endpoint (August 1, 2020) based on any contact with the patient. Patients who did not progress by the study endpoint were censored based on the last clinical assessment.

Of the study cohort, 27 patients had genetic testing of metastatic disease preceding SABR with an institutional targeted sequencing platform that examines up to 468 genes^13^, ^14^ (Table S8). The presence of any mutation within a gene exon or a copy number change was noted for each of the 27 patients. We compared 13 pathways from the Kyoto Encyclopedia of Genes and Genomes (KEGG) with observed gene alterations within our sample to identify altered pathway‐level effects involving at least two patients.^15^

### 2.2 Statistics

Overall survival (OS) was defined as the time from the beginning of SABR to death or censoring at the date of last patient contact. Progression‐free survival (PFS) was defined as the time from the beginning of SABR until progression of disease, death, or censoring at the date of last patient clinical assessment. We defined the progression of disease locally and systemically using the modified RECIST and PERCIST criteria.^16^ Time to local progression (TTLP) was calculated as the time from the beginning of SABR until the progression of disease within the SABR field or immediately adjacent area, even if a patient was noted to progress systemically at a prior date. Likewise, time to distant metastatic progression (TTDM) was calculated as the time from the beginning of SABR to the development of out of RT field disease, even if a patient was noted to have prior local progression of disease. The time to subsequent therapy (TTST) was defined as the time from the start of SABR to the next radiotherapy or systemic therapy, excluding any systemic therapy that started concurrently with SABR.

The number of metastases treated at baseline was compared with the presence of symptoms and toxicity using Fisher's exact test. An unbiased estimate of median follow‐up time was obtained using the reverse Kaplan–Meier method with the interquartile range (IQR) reported. The number of treated sites and time from diagnosis to SABR were dichotomized using their medians. Through Kaplan–Meier analysis, estimates of PFS, OS, TTLP, TTST, and TTDM were obtained, and a log‐rank test was used to examine univariate (UVA) correlations with OS, PFS, and TTDM after SABR. In addition, UVA Cox proportional hazards and multivariate (MVA) Cox proportional hazards regression models were used to derive effect sizes and determine independent associations with OS, PFS, and TTDM after SABR. For each independent variable in the models, we report the effects (i.e., hazard ratio) and their associated significance. The assumption of proportional hazards was verified with univariate Cox proportional hazards regression models, and the final MVA model was stratified by any effects shown to violate proportional hazards assumptions during UVA. For these effects, we report the median time to outcomes. Because progression of disease at distant sites, driving both TTDM and PFS in the sample, nearly always occurred before death, death was considered as a competing event only for TTST and TTLP. We used competing risk analysis to determine the cumulative incidence of TTST, TTLP, and OS separately for correlates, and we report chi‐square tests of significance. UVA and MVA included molecular subtype, AJCC 8^th^ edition staging, T stage of primary disease, N stage of primary disease, age at SABR, time from diagnosis to SABR, site of OM, type of OM, number of OM treated, and the number of chemotherapies before SABR. Additionally, for TTLP, we considered the importance of radiation dose. A significance threshold of 0.05 was used for statistical modeling, and in the case of genetic analysis with numerous comparisons, false discovery rates (fdr) <0.05 were considered to account for multiple testing. All statistical analyses were performed using R.

## 3 RESULTS

### 3.1 Patient characteristics

**FIGURE 1:**
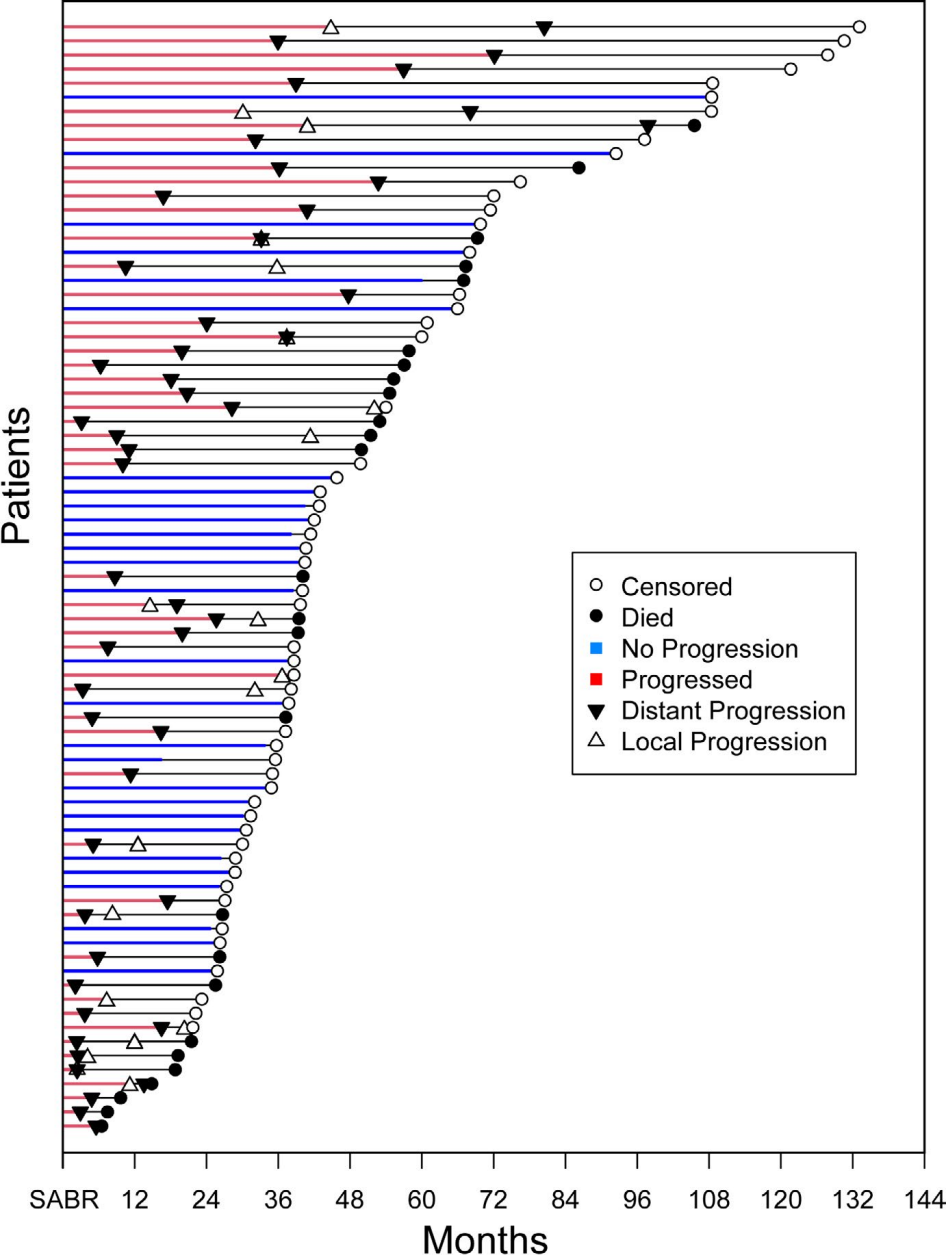
Natural history of disease progression following SABR. In this swimmer plot, each patient is represented as a black line indicating the time from SABR until outcomes of interest. The time to the first progression is shown for patients who progressed (red) and those who did not progress (blue). For those patients who progressed, distant progression (black triangle) and local progression (white triangle) are shown. For all patients, death (black circle) and censoring (white circle) are also indicated. Of those who died, all patients except one were noted to have distant progression prior to death, indicating that death from any cause is not major a competing event for observing distant progression; whereas, many patients did not have observed local progression before death

Table 1 details patient and tumor characteristics. The median patient age at SABR for OM was 56 years (30–83 years). A majority of patients (n=66, 84%) had HR+/HER2‐ breast cancer, eight (10%) had HER2+ breast cancer, and five (6%) had triple‐negative (TNBC) breast cancer. Most patients (80%) received SABR to 1 OM with osseous metastases as the most common site. Figure S1 shows the pre‐RT imaging, plan, and post‐RT imaging of a patient treated for a bleeding chest nodule. Thirty‐five (44%) patients underwent SABR for newly diagnosed metastatic disease, 7 (9%) for stable metastatic disease on systemic therapy, and 37 (47%) for progressing oligometastatic disease. Median follow‐up was 50 months (IQR: 29–66 months) with 53 (67%) patients alive and 51 (65%) patients with progression of disease at the last follow‐up. Of the patients who progressed, most (n = 42, 82%) developed new metastases and nine (18%) patients experienced local failure at the SABR site. At the time of progression, of 51 patients who progressed, 41 (80%) patients progressed at 5 or fewer sites, and the OM was treated with radiotherapy in 9 (18%) patients. Of 41 patients with oligoprogression, 49 sites of progression were noted with 29 (59%) as bone metastases, 7 (14%) lymph node metastases, 4 (8%) liver metastases, 3 (6%) soft tissue metastases, 5 (10%) lung nodules, and 1 (2%) brain metastasis (Table 2). A swimmer plot summarizing the study findings is shown in Figure 1.

**TABLE 1:** Patient and tumor characteristics

Characteristics	N(%)
Sex	
Female	77 (97%)
Male	2 (3%)
Age (Median 56; range 30–83)	
30–49	26 (33%)
50–64	33 (42%)
65–83	20 (25%)
AJCC Stage at diagnosis	
I	16 (20%)
II	30 (38%)
III	21 (27%)
IV	12 (15%)
Pathologic T Stage of primary tumor	
T1/T2	70 (89%)
T3/T4	9 (11%)
Pathologic N Stage of primary tumor	
N0/N1mic	35 (44%)
N1a/N2a/N3a	44 (56%)
Tumor Subtype	
HR+/HER2‐	66 (84%)
HR+/HER2+	7 (9%)
HR‐/HER2+	1 (1%)
TNBC	5 (6%)
Type of OM	
Newly diagnosed OM	35 (44%)
Stable OM	7 (9%)
Progression of known OM	37 (47%)
Time from diagnosis to SABR (Median 5 years; range 0–25)	
<5 year	39 (49%)
≥5 years	40 (51%)
Lines of systemic therapy before SABR for OM	
0	16 (20%)
≥1	63 (80%)
Number of treated metastases per patient	
1	63 (80%)
≥1	16 (20%)
Metastatic sites (n=103)	
Bone	96 (93%)
Lymph nodes	4 (4%)
Lung	2 (2%)
Skin	1 (1%)
SABR Dose Fractionation (n=103)	
18–24 Gy x 1	32 (31%)
8–10 Gy x 3	43 (42%)
10–12 Gy x 4	3 (3%)
5–7 Gy x 5	22 (21%)
5 Gy x 8	3 (3%)

**TABLE 2:** Details of patient progression after SABR

Progressed (n=51)	n (% of 51)
Unplanned radiotherapy at progression	9 (18%)
Unplanned systemic therapy at progression	41 (80%)
No therapy at first progression	1 (2%)
Local	9 (18%)
Distant	42 (82%)

Oligoprogression (n=41)	n (% of 41)
Oligoprogression treated with radiotherapy	7 (17%)
Oligoprogression treated with systemic therapy	34 (83%)

Sites of oligoprogression (n=49)	n (% of 49)
Bone	29 (59%)
Spine	11 (22%)
Pelvic bones	7 (14%)
Rib	4 (8%)
Humerus	2 (4%)
Sternum	2 (4%)
Calvarium	1 (2%)
Femur	1 (2%)
Scapula	1 (2%)
Non‐bone	20 (41%)
LN	7 (14%)
Lung	5 (10%)
Liver	4 (8%)
Soft tissue	1 (2%)
Omentum	1 (2%)
Brain	1 (2%)
Breast	1 (2%)

### 3.2 Overall and progression‐free survival

**FIGURE 2:**
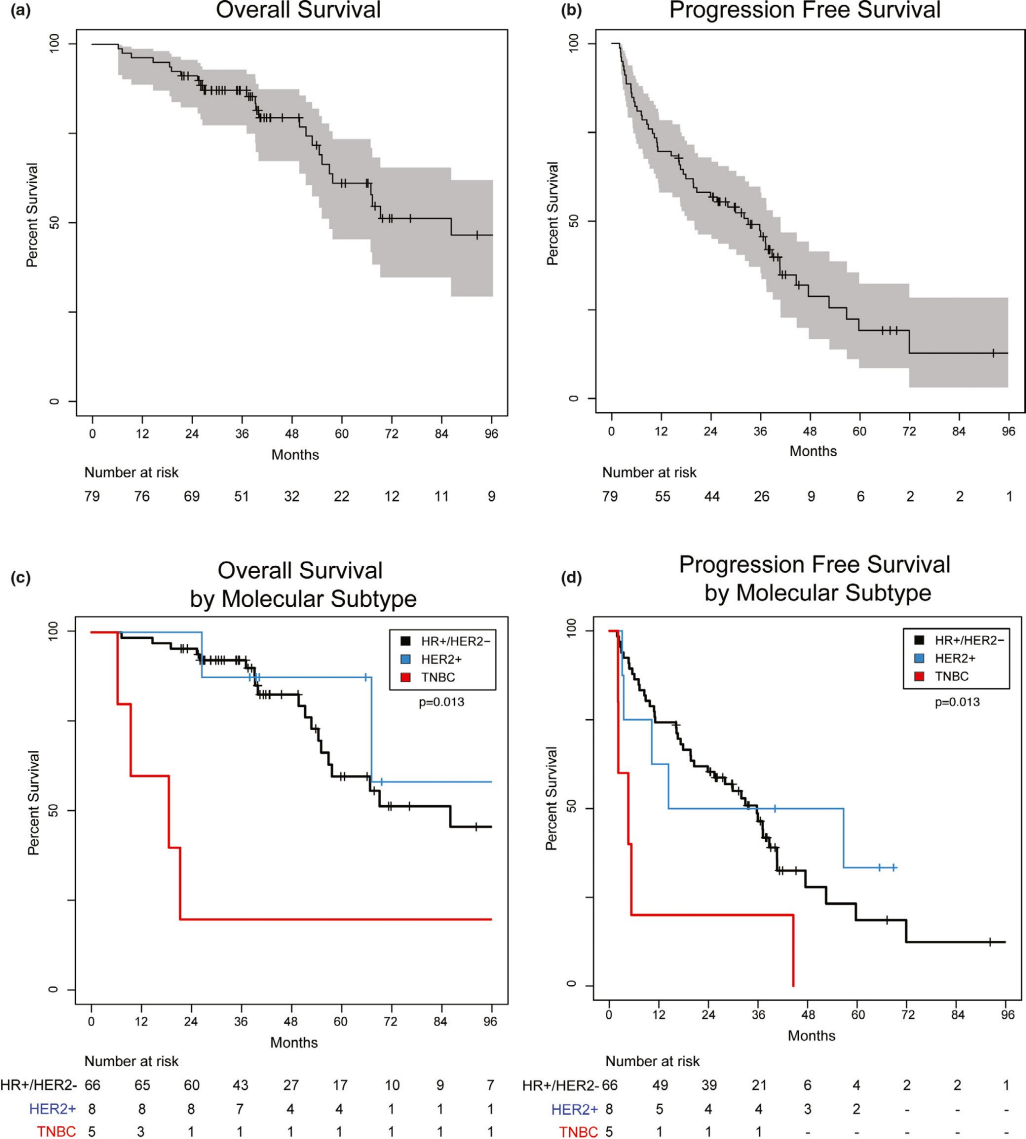
Overall survival (OS) and progression‐free survival (PFS) by molecular subtype. A) Kaplan–Meier analysis of OS for the entire cohort demonstrates a median OS of 86 months (IQR: 29–66 months). A 95% confidence band is shown in gray. B) PFS for the entire cohort is shown with a median PFS of 33 months (IQR: 10–38 months). A 95% confidence band is shown in gray. C) OS is shown stratified by molecular subtype. HR+/HER2‐, HER2+, and TNBC had significantly different overall survival [log‐rank *p*=0.013]. Median OS for HR+/HER2‐, HER2+, and TNBC was 86 months (IQR: 31–60 months), 57 months (IQR: 9–59 months), and 18 months (IQR: 9–21 months), respectively. D) PFS is shown stratified by molecular subtype [log‐rank *p*=0.013]. The median PFS for HR+/HER2‐, HER2+, and TNBC was 36 months (IQR: 12–38 months), 57 months, (IQR: 9–59 months), and 5 months (IQR: 2–5 months), respectively

**FIGURE 3:**
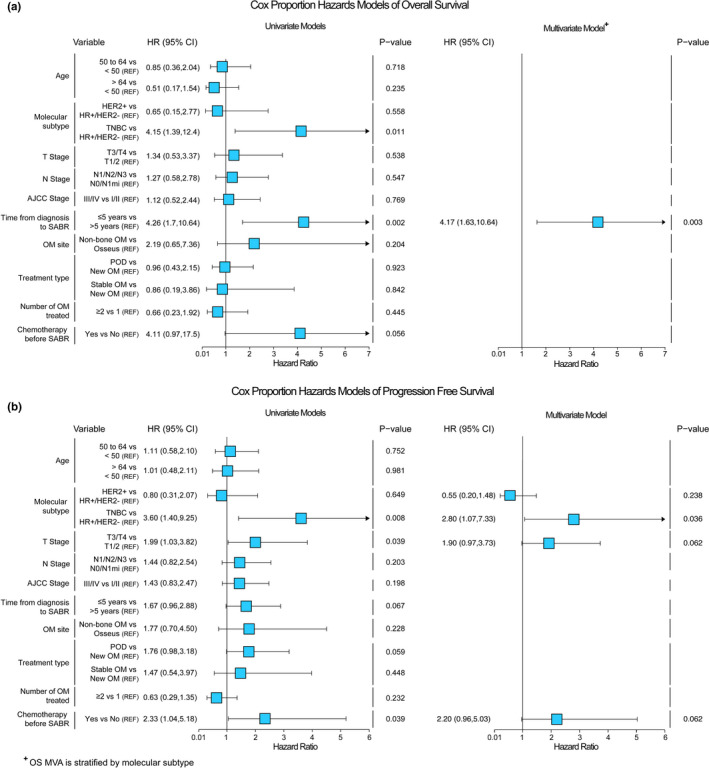
Forest plots indicating univariate (UVA) and multivariate (MVA) analyses for A) overall survival (OS) and B) progression‐free survival (PFS). The hazard ratios (HR) and 95% confidence intervals (CI) are shown. The reference (REF) categories for hazard ratios are indicated. OM refers to oligometastases. Only factors found to be significant in the UVA were included in the Cox proportional hazard MVA. For OS, the final model was stratified by molecular subtype and less time from diagnoses to SABR associated with worse OS. For PFS, having TNBC was associated with worse PFS compared to HR+/HER2‐ breast cancer

We observed a median OS of 86 months (IQR: 29–66 months) (Figure 2A). The median OS for patients by molecular subtype was 86 months (IQR: 31–60 months) for HR+/HER2‐ breast cancer, not reached (IQR: 39–67 months) for HER2+, and 18 months (IQR: 9–21 months) for TNBC (Figure 2C). On UVA, molecular subtype (*p *= 0.020) and <5 years from breast cancer diagnosis to SABR (*p* < 0.001) were associated with shorter OS (Figure 3A). Because proportional hazards assumptions did not hold for molecular subtype and OS, the MVA was stratified by molecular subtype, and <5 years from breast cancer diagnosis to SABR (*p *= 0.004) was associated with shorter overall survival (Figure 3A).

We observed a median PFS of 33 months (IQR: 10–38 months) (Figure 2B). On UVA, molecular subtype (*p *= 0.01), any chemotherapy prior to SABR (*p* < 0.001), and T3/T4 at diagnosis (*p *= 0.040) were associated with shorter PFS (Figure 3B). The median PFS for patients by molecular subtype was 36 months (IQR: 12–38 months) for HR+/HER2‐ breast cancer, 57 months (IQR: 9–59 months) for HER2+, and 5 months (IQR: 2–5 months) for TNBC (Figure 2D). On MVA, only having TNBC relative to HR+/HER2‐ (*p *= 0.036) was associated with shorter PFS (Figure 3B).

We did not find a significant difference in OS or PFS for patients who underwent SABR for newly diagnosed OM, stable OM on systemic therapy, or progressing OM. We considered whether lines of endocrine therapy or the combined use of chemotherapy and endocrine therapy, that is, total systemic therapy lines, were associated with OS or PFS. Though total systemic therapy lines were associated with PFS, we did not find endocrine therapy before SABR was associated with either outcome. Therefore, the effect of total systemic therapy was driven by chemotherapy use.

### 3.3 Time to subsequent therapy, time to local progression, and time to distant metastases

The median TTST was 28 months (IQR: 10–40 months). The median TTLP was not observed over the study period but the IQR was 26–54 months. On UVA using competing risk analysis, no factors were significantly associated with TTST or TTLP. The median TTDM was 36 months (IQR: 11–39 months). Having any chemotherapy prior to SABR (*p* = 0.040, HR = 2.3 [1.0, 5.2]) and having T3‐T4 disease at diagnosis (*p *= 0.030, HR = 2.0 [1.9, 2.2]) were associated with shorter TTDM, as was the case with models of PFS; however, <5 years between cancer diagnoses and SABR (*p *= 0.050, HR = 1.9 [1.1–3.4) was also associated. In MVA, the factors predicting TTDM were not significant when controlling for one another.

### 3.4 Symptom assessment and toxicity

Symptom assessment and toxicity of radiotherapy are shown in Table 3. At the time of SABR, 52 (66%) of patients reported symptomatic oligometastases with 46 (88%) of the symptomatic patients reporting pain. There were 13 (16%) patients with a pathologic fracture at baseline, 5 of whom had planned stabilization surgery prior to or immediately after radiotherapy. While patients who had >1 treated oligometastases were significantly more likely to have symptoms at baseline (Fisher's exact *p *= 0.008), they were not more likely to have any acute (Fisher's exact *p *= 0.099), subacute (Fisher's exact *p *= 0.211), or chronic (Fisher's exact *p *= 0.999) treatment‐related toxicity compared to those with one treated oligometastasis. During radiotherapy and the following 2 weeks, 39 (49%) patients reported acute toxicity. Of patients reporting toxicity, pain flare requiring steroids was seen in 9 (23%) patients, fatigue was reported in 21 (54%) patients, and skin reactions were seen in 9 (23%) patients. During the 2 to 6 months following radiotherapy, 46 (88%) patients reported the improvement of their symptomatic metastasis after radiotherapy. In the subacute phase, 58 (73%) patients reported any new, continued, or worsening symptom where attribution to radiotherapy could not be excluded including pain in 35 (60%) patients, musculoskeletal symptoms in 19 patients (33%), gastrointestinal symptoms in 15 (25%) patients, skin symptoms in 14 (24%) patients, and lung symptoms in 5 (9%) patients. In the late phase, 14 patients reported worsening pain at the site of treated oligometastasis, 11 (15% of bone radiotherapy recipients) patients experienced and in‐field pathologic fracture, and 1 patient experienced radiation pneumonitis. No chronic gastrointestinal complications could be attributed to in‐field radiotherapy effects.

**TABLE 3:** Patient symptoms and toxicities by category and timepoint. Having any symptom is reported as a percentage of the total number of patients (n=79). Each symptom is reported as a percentage of patients reporting any symptom at a timepoint. MSK, musculoskeletal. GI, gastrointestinal

	Toxicity/Symptom	Baseline	Acute (on RT to 2 weeks post‐RT)	Subacute (2 to 6 months post‐RT)	Late (after 6 months)
		n (%)	n (%)	n (%)	n (%)
Any		52 (66%)	39 (49%)	58 (73%)	23 (29%)
Pain	Pain	46 (88%)	6 (15%)	35 (60%)	14 (61%)
	Pain flare requiring steroids	‐	9 (23%)	‐	‐
	Worsening neuropathy	‐	‐	3 (5%)	‐
	Headache	1 (2%)	‐	1 (2%)	‐
Skin	Dermatitis	‐	9 (23%)	11 (19%)	1 (4%)
	Bleeding	1 (2%)	1 (3%)		
	Hyperpigmentation	‐		1 (2%)	‐
MSK	Fatigue	‐	21 (54%)	7 (12%)	‐
	Numbness/paresthesia	3 (6%)	‐	5 (9%)	‐
	Myositis		‐	4 (7%)	3 (13%)
	Muscle tightness		‐	1 (2%)	‐
	Weakness	2 (4%)	‐	1 (2%)	‐
	Edema	‐	‐	2 (3%)	1 (4%)
	Fibrosis	‐	‐	1 (2%)	‐
	Limited range of movement	‐	‐	1 (2%)	‐
	Arthralgias	‐	‐	1 (2%)	‐
	Muscle spasms	‐	‐	1 (2%)	‐
	Osteonecrosis of the jaw	‐	‐	‐	1 (4%)
	Pathologic fracture	25 (16%)	‐	‐	11 (48%)
	Stabilization surgery	‐	‐	‐	10 (43%)
Lung	Dyspnea	1 (2%)	1 (3%)	1 (2%)	
	Cough	1 (2%)	‐	5 (9%)	‐
	Chest tightness	1 (2%)	‐	1 (2%)	‐
	Pneumonitis		‐	1 (2%)	2 (9%)
GI	Esophagitis		2 (5%)	13 (22%)	‐
	Nausea		9 (23%)	2 (3%)	‐
	Diarrhea		1 (3%)	2 (3%)	‐
	GERD		‐	1 (2%)	‐

### 3.5 Genetic correlates

**FIGURE 4:**
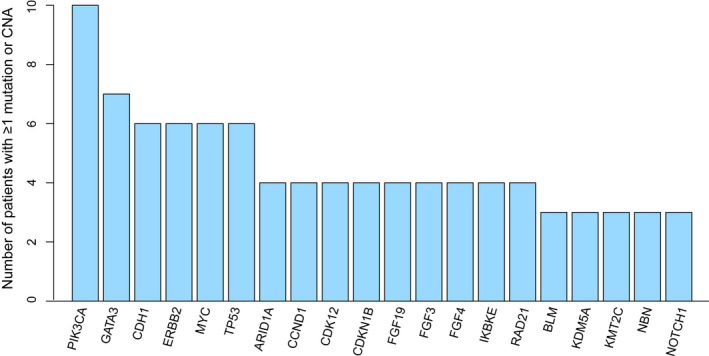
The 20 most frequently altered genes in the metastases of the subset of oligometastatic breast cancer patients who had target sequencing (n=27). Alterations include mutations and copy number aberrations (CNA). PIK3CA is the most frequently altered gene

Of the 27 patients with targeted sequencing of metastases, 10 (37%) patients had an alteration in PIK3CA including 5 patients with a specific H1047K mutation in PIK3CA, 3 patients with E545K mutation, and 3 patients with more than 1 gene alteration. Other commonly altered genes were GATA3 occurring in seven (26%) patients, and ERRB2, TP53, CDH1, and MYC alterations each occurring in six patients (22%) (Figure 4). Mutations or copy number changes in CEBPB (7%), RB1 (7%,), TBX3 (11%), PTEN (7%) or CDK4 (7%) were associated with different clinical outcomes. Alterations of CEBPB, RB1, TBX3 and CDK4 were associated with shorter PFS (fdr = =2 × 10^−7^, fdr = 2 × 10^−7^, fdr = 4 × 10^−5^, and fdr = 0.003, respectively) and shorter TTDM (fdr = 2 × 10^−7^, fdr = 2 × 10^−7^, fdr = 4 × 10^−5^, and fdr = =0.003, respectively). Alterations in CEBPB, RB1, CDK4, and PTEN were associated with shorter OS (fdr = 3 × 10^−5^, fdr = 3 × 10^−5^, fdr = 3 × 10^−4^ and fdr = 0.014, respectively) and alterations of RB1, CDK4, and PTEN were associated with shorter TTLP (fdr = 0.008, fdr = 0.008, and fdr = 0.008, respectively). At the pathway level, alterations in the KEGG DNA replication pathway were associated with shorter PFS (fdr = 0.025). The genes altered in this pathway include DNA polymerase epsilon (POLE) and DNA polymerase delta 1 (POLD1), altered in a TNBC patient and an HR+/HER2+ patient, respectively.

## 4 DISCUSSION

This is one of the largest retrospective series to date examining the population of breast cancer OM patients treated with SABR for extracranial metastases, and by only including patients without prior RT for extracranial metastatic disease, requiring a high BED_4_ treatment to all OM sites, having a long follow‐up time allowing us to report treatment complications, incorporating tumor genetics, and characterizing disease progression, we believe that this study adds clinically important data to the current breast cancer OM literature. We found that patients had a relatively long median OS of 86 months and PFS of 33 months. Our results compared favorably to previous studies of SABR for OM in breast cancer. In a 40‐patient study, Milano et al., 2009, found 4‐year actuarial OS and PFS to be 59% and 38%, respectively,^17^ compared to 4‐year OS and PFS in our study of 79% and 29%, respectively (Table S7). In 2018, Trovo et al. reported 2‐year OS and PFS of 95% and 53%, respectively, in a prospective study^18^ which is similar to the 2‐year OS and PFS of 91% and 57%, respectively, observed in our study (Table S7). We determined that patients had excellent local control after SABR, even when they progressed at distant sites. With a median follow‐up of 50 months, the median time to local progression (TTLP) was not reached. The reported 4‐year local control of breast cancer oligometastases treated with SABR was estimated to be 89%,^17^ and our study estimate of 4‐year local control was comparable at 70% (95% CI: 58%–83%). In addition, we found that most patients (82%) have oligoprogression at a distant site when they progress, but a majority of patients received only systemic therapy instead of additional local therapy. Though reporting of patterns of failure from other studies of SABR and breast cancer with OM confirms that distant metastases occur much more often than local recurrence, the rate of oligoprogression is generally not reported.^18^, ^19^ In studies of prostate cancer with OM treated with SABR, approximately only 25% of patients have oligoprogression.^20^ Thus, our finding of a high number of oligoprogressive lesions in breast cancer patients with OM after SABR suggests the possibility of receiving additional local therapy to new sites of disease and potentially achieving longer disease control or survival benefit.

Our results also compared favorably to studies of alternative therapies for OM in breast cancer. When compared a similar population of HR+/HER2‐ patients who were eligible for local therapy for OM, but instead only received chemotherapy without local control, the median PFS for paclitaxel^21^, ^22^ or docetaxel,^23^ antimicrotubular antineoplastics/capecitabine,^21^, ^24^ docetaxel/sunitinib,^23^ doxorubicin,^22^ doxorubicin/paclitaxel^22^ ranged between 6 and 10 months, though the majority of the patients in our cohort did not receive these specific agents concurrently with SABR. A large number (n = 46 [62%]) of patients in our cohort received hormone therapy as their only concurrent systemic agent with SABR (Table S5), and hormone therapy alone without local therapy is estimated to have a PFS between 4.6 and 14.7 months.^25^ Likewise, 17 (23%) of the patients in our cohort received Palbociclib and hormone therapy with SABR (Table S5), and estimates show PFS without local therapy ranges from 9.2 to 24.8 months.^26^, ^27^ Taken together, these estimates underscore a potential role for SABR in increasing PFS in the setting of a concurrent systemic agent. When compared to OM breast cancer patients who received metastasectomy of their non‐osseous disease, patients with liver resection had a 5‐year overall survival of 48.4% and a 3‐year disease‐free survival rate of 46%,^28^ while patients who received lobectomy with systemic therapy had a 3‐year PFS 55% and 4‐year OS at 82%,^29^ both of which are comparable to our study (Table S7). Metastasectomy for skeletal metastases has shown a post‐operative median survival for patients with skeletal breast metastases as low as 8 months^30^ which is significantly worse than our cohort of patients with primarily osseous OM, but this may reflect differences in the severity of disease for patients who are referred for metastasectomy.

We found that patients with oligometastatic TNBC tend to have worse OS and PFS compared to patients with oligometastatic HR+/HER2‐ breast cancer; however, only five patients had TNBC, so the effects of SABR for OM in this population are still unclear. Independent of molecular subtype, time from initial breast cancer diagnosis to SABR significantly predicted OS, and T stage at diagnosis, prior chemotherapy, and a short interval from diagnosis to SABR were associated with outcomes in our patient cohort. These clinical factors are related to one another and likely reflect the aggressiveness of the disease as patients with shorter onset to develop metastatic disease have had more systemic progression history and may not benefit as much from SABR compared to those with more indolent disease course. This observation is consistent with Milano et al., 2012, finding that radiographic progression after systemic therapy but before SABR was associated with worse OS.^19^ Interestingly, we did not find that oligometastatic disease state (i.e., oligometastatic at metastatic presentation, progression of known metastasis, or stable oligometastasis), which should be a surrogate for different levels of disease aggressiveness, predicted outcomes. We also did not find any association with either OS or TTDM and the number of OM or sites of treated OM as reported in a recent study.^17^ Last, we did not find any association between BED_4_ and TTLP as previously reported^31^; however, we limited our study inclusion criteria to only include patients with a BED_4_ ≥60 Gy, which more accurately reflects SABR treatment. We may have been underpowered to detect these differences as was the case in prior studies.^18^

There were important toxicities observed both during and after radiotherapy. While on treatment, of symptomatic patients, 38% experienced worsening pain or a pain flare requiring steroids, 54% experienced fatigue, and 23% experienced nausea, all of which are expected side effects of radiotherapy and can be treated supportively.^32^ In the subacute setting, of the patients who experienced symptoms, 60% experienced pain, 31% experienced dermatitis or hyperpigmentation, and 30% experienced GI symptoms; however, 88% of patients who were symptomatic at baseline reported the improvement of their symptoms, consistent with prior reports.^33^ Likewise, in the late effect setting, only 23 patients had complaints potentially related to their treated lesion, with 14 patients reporting continued pain, 11 patients having pathologic fracture with 10 requiring surgery, and 2 patients with pneumonitis attributed to radiotherapy. Of the 71 bone lesions not associated with pathologic fracture before SABR, 11 (15%) eventually had a pathologic fracture, concordant with a known risk of late pathologic fracture following radiotherapy for bone metastases as high as 20%,^34^ but the presence of a tumor, patient age, and concurrent systemic agents can make the contribution of SABR difficult to measure. Likewise, late radiation pneumonitis is a known and potentially serious consequence of lung irradiation which can be minimized by SABR relative to other types of radiotherapy and is usually treatable with steroids.^35^ Patients should be counseled on these potentially serious consequences of SABR in the setting of the potential for symptomatic relief, improved local control, and potential improved PFS and OS.

We performed an exploratory genomic analysis in a subset of patients and found associations of outcomes with several genes. We observed that breast cancer oligometastases mostly commonly had alterations of PIK3CA (37% in our cohort), GATA3, ERRB2, TP53, CDH1, and MYC. In metastatic breast cancer, PIK3CA, GATA3, ERRB2, TP53, and CDH1 are commonly mutated.^36^ Though MYC is less commonly altered in breast cancer, it is a well‐known oncogene and has been associated with metastatic disease.^37^ We found that mutations and copy number changes in CEBPB, RB1, TBX3, PTEN, and CDK4 were associated with worse survival outcomes. Through pathway analysis, we also determined that alterations in the KEGG DNA replication pathway correlate with worse PFS. There is evidence that mutations in the replication pathway can lead to a microsatellite unstable phenotype, which could provide a basis for metastatic change or resistance to radiotherapy.^38^ It is possible that alterations of these genes may indicate altered biology of the breast cancer oligometastases with a worse response to radiotherapy, but adequately powered studies are needed to validate these findings. RB1 mutation is a known primary and acquired resistance mechanism to CDK4/6 inhibitors in HR+metastatic breast cancer, which could be a confounding factor in our analysis.^39^ Because 17 patients (23%) received CDK4/6‐directed therapy concurrently with SABR, these therapies are potentially safe to give with SABR and may contribute to improved outcomes.

There are several limitations to our analysis. Given that our cohort is one of the largest to date, we aimed to perform multiple analyses and obtain estimates at the cost of a high type I error, and, therefore, we recommend that the study associations should be validated through prospective studies. Also, due to the retrospective nature of this study, we are unable to account for all possible confounding variables that may influence patient outcomes, and we could not ensure the completeness, attribution, or grade of symptom and toxicity data. Our cohort is possibly positively selected for better outcomes as the majority of patients had 1 OM and had osseous OM. Although our findings were independent of these factors, this has implications for generalizability. Even with this relatively large retrospective cohort of breast cancer OM patients treated with SABR, there still may not be a sufficient number of patients in our study to determine statistically significant associations of clinical correlates like radiation dose and disease outcomes. Our median follow‐up was 50 months, and for HR+/HER2‐ metastatic breast cancer, longer follow‐up time may be needed to model overall survival. Last, only a subset of patients had genomic testing, and a larger cohort is needed to validate the interesting genetic predictors determined in our study.

## 5 CONCLUSION

We have shown that long‐term disease control and survival can be achieved with SABR for oligometastatic breast cancer. These findings suggest that select patients with hormone receptor‐positive breast cancer with oligometastatic disease, especially those who presented with early T stage, had a long disease‐free interval from initial diagnosis, and had a limited systemic progression history, may be considered for SABR to all sites of disease. Further studies are needed to clarify the role of SABR for breast cancer with OM in TNBC. There may be genetic factors that can distinguish OM from primary breast cancer and polymetastatic patients as well as responders to therapy, but this remains to be elucidated.

## CONFLICT OF INTEREST

The authors have no disclosures of COI related to this study or its subject matter.

## AUTHOR CONTRIBUTIONS

JTY and AJX devised the project, the main conceptual ideas, and helped to write the manuscript. NAW conducted record review, performed statistical analysis, and helped to write the manuscript. CDA devised the project, conducted record review, and helped to write the manuscript. LD helped to write the manuscript. CJT helped to write the manuscript. EG helped to write the manuscript. WIZ helped to write the manuscript. MR and YY provided expert support in topics discussed in the manuscript.

The de‐identified data that support the findings of this study are available in supplemental tables or upon request to the corresponding author. The entire dataset is not publicly available due to privacy and ethical restrictions.

## Supporting information

Fig S1

Table S1‐S8
