# Supplementary material for: Long‐term disease control and survival observed after stereotactic ablative body radiotherapy for oligometastatic breast cancer
Source: Cancer Med. 2021 Jun 22;10(15):5163–74. doi: 10.1002/cam4.4068 (PMC8335830; doi:10.1002/cam4.4068)
Supplement: Supplementary file 1 — Fig S1 [file CAM4-10-5163-s002.docx]

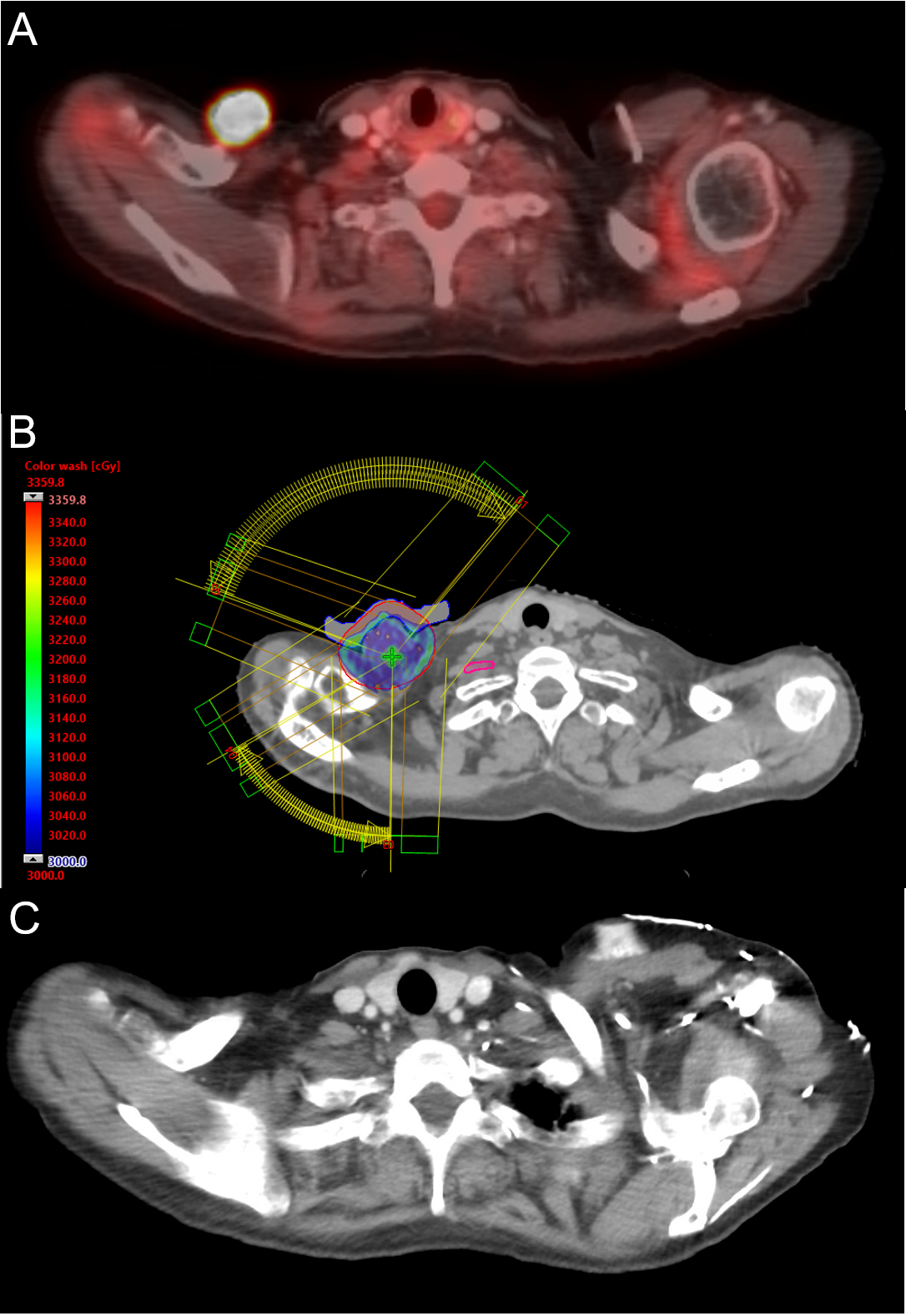


Figure S1. A) 56-year-old F with oligometastatic breast cancer presenting with a symptomatic (bleeding) right chest wall lesion. B) The lesion was treated with 30Gy in 5 fractions SABR with a bolus. C) On treatment, she reported dermatitis and worsening of the mass bleeding, but these symptoms began to improve by 1 month post radiotherapy. At 3 month post-radiotherapy scan, the lesion has shown a good response to SABR with improvement of symptoms.
